# Lifestyle factors and lumbar disc disease: results of a German multi-center case-control study (EPILIFT)

**DOI:** 10.1186/ar3164

**Published:** 2010-10-18

**Authors:** Barbara Schumann, Ulrich Bolm-Audorff, Annekatrin Bergmann, Rolf Ellegast, Gine Elsner, Joachim Grifka, Johannes Haerting, Matthias Jäger, Martina Michaelis, Andreas Seidler

**Affiliations:** 1 Federal Institute for Occupational Safety and Health, Nöldnerstraße 40-42, 10317 Berlin, Germany; 2 Institute f. Med. Epidemiology, Martin-Luther-University Halle-Wittenberg, Magdeburger Straße 27, 06097 Halle/Saale, Germany; 3 Labor Inspection, Occupational Health Division, Simone-Veil-Straße 5, 65197 Wiesbaden, Germany; 4 Institute for Occupational Safety and Health of the German Social Accident Insurance (IFA), Alte Heerstraße 111, 53757 St. Augustin, Germany; 5 Institute of Occupational Medicine, Johann Wolfgang Goethe-University, Theodor Stern-Kai 7, 60590 Frankfurt am Main, Germany; 6 Orthopaedic Clinic, University of Regensburg, Kaiser-Karl-V-Allee 3, 93077 Bad Abbach, Germany; 7 Leibniz Research Centre for Working Environment and Human Factors, Dortmund University of Technology, Ardeystraße 67, 44139 Dortmund, Germany; 8 Research Center for Occupational and Social Medicine Freiburg, Bertoldstraße 27, 79098 Freiburg, Germany; 9 Institute and Policlinic for Occupational and Social Medicine, Fetscherstr. 74, 01307 Dresden, Germany

## Abstract

**Introduction:**

In the large-scale case-control study EPILIFT, we investigated the dose-response relationship between lifestyle factors (weight, smoking amount, cumulative duration of different sports activities) and lumbar disc disease.

**Methods:**

In four German study regions (Frankfurt am Main, Freiburg, Halle/Saale, Regensburg), 564 male and female patients with lumbar disc herniation and 351 patients with lumbar disc narrowing (chondrosis) aged 25 to 70 years were prospectively recruited. From the regional population registers, 901 population control subjects were randomly selected. In a structured personal interview, we enquired as to body weight at different ages, body height, cumulative smoking amount and cumulative duration of different sports activities. Confounders were selected according to biological plausibility and to the change-in-estimate criterion. Adjusted, gender-stratified odds ratios with 95% confidence intervals were calculated using unconditional logistic regression analysis.

**Results:**

The results of this case-control study reveal a positive association between weight and lumbar disc herniation as well as lumbar disc narrowing among men and women. A medium amount of pack-years was associated with lumbar disc herniation and narrowing in men and women. A non-significantly lowered risk of lumbar disc disease was found in men with high levels of cumulative body building and strength training.

**Conclusions:**

According to our multi-center case-control study, body weight might be related to lumbar disc herniation as well as to lumbar disc narrowing. Further research should clarify the potential protective role of body building or strength training on lumbar disc disease.

## Introduction

Disc-related diseases of the lumbar spine, such as lumbar disc herniation and disc narrowing, are common health problems and frequently lead to work disability. In addition to established risk factors such as high-impact mechanical pressure due to manual materials handling, inflammatory and atherosclerotic processes are discussed. Thus, cardiovascular risk factors and unhealthy lifestyle-related factors (for example, excess weight, smoking, and physical inactivity) potentially constitute risk factors for lumbar disc disease; however, empirical evidence remains controversial. In a recent systematic review of 25 papers on arteriosclerosis and disc degeneration/lower back pain [1], it was concluded that in cohorts of older people and in large study samples, weak but consistent associations between smoking and other cardiovascular risk factors and disc degeneration and lower back pain could be determined. However, the methods, study population, and results of these studies differ to a considerable degree and so, to date, no reliable conclusions have been drawn. The German Spine Study EPILIFT investigated the association between cumulative workload and lumbar disc diseases. The advantages of the study are a sufficient sample size and x-ray-confirmed diagnoses of lumbar disc herniation and lumbar disc narrowing. Occupational history and non-occupational risk factors were assessed in detail. The aim of the present analysis was to assess the dose-response relationship between lifestyle-related factors (smoking, body mass index [BMI], and sports activities) and lumbar disc disease (lumbar disc herniation and lumbar disc narrowing) on the basis of data of the EPILIFT Study.

## Materials and methods

Details about the design of the EPILIFT study have been published elsewhere [2,3]. In brief, the EPILIFT study is a population-based, multi-center, case-control study performed in four German study regions: Frankfurt/Main, Freiburg, Halle/Saale, and Regensburg.

### Study population

All male and female patients who were 25 to 70 years old and who were treated in the mentioned regions were included if they fulfilled the following criteria (1 or 2): they were (1) in- or outpatients of participating hospitals and received treatment because of lumbar disc herniation with radiculopathy with sensory or motor deficits (neurological findings) or they were (2) in- or outpatients of participating hospitals or outpatients of participating orthopedic practices and received treatment because of severe disc space narrowing (chondrosis) with radiculopathy with sensory or motor deficits or local lumbar syndrome.

The diagnosis of lumbar disc herniation had to have been confirmed by computerized tomography (CT) or by magnetic resonance imaging (MRI); the diagnosis of lumbar disc narrowing was based primarily on x-rays. Disc herniation and disc space narrowing were defined as proposed by a consensus group [4]. Altogether, 1,112 patients were reported by the participating physicians. Finally, to determine which patients qualified as cases, a reference radiologist reassessed MRI, CT, and x-rays of the lumbar spine individually for each disc and vertebral body. Furthermore, the clinical diagnosis had to be verified by an experienced reference orthopedist. As a result of this diagnostic verification, 197 patients did not fulfill the inclusion criteria and were excluded from the study. In total, 915 cases were included (286 men and 278 women with lumbar disc herniation and 145 men and 206 women with lumbar disc space narrowing). The response rate was 66.4% among the cases. Control subjects were randomly selected from a 1% random sample of residents who were 25 to 70 years old and who were selected by the local population registration offices of the same regions. Of the 1,687 population controls, 901 (53.4%) agreed to participate. According to a non-responder analysis, the proportion of blue-collar workers was higher among non-responding cases and among non-responding control subjects. We found no evidence for a differential selection bias with respect to social status.

### Exposure assessment

Trained interviewers conducted a detailed computer-assisted personal interview in regard to work-time physical workload (including lifting and carrying of loads, pushing, digging, working postures, and whole-body vibration), psychosocial workload, weight at different ages since adulthood, height, smoking history, sports activities, leisure activities, and critical life events. Psychosocial stress at work was assessed with the German screening tool 'FIT', based on Karasek's job strain model [5,6]. With subjects exceeding a certain workload level, a semi-standardized expert interview was performed, documenting the intensity, frequency, and duration of all spine-related exposures induced by manual materials handling, trunk inclination and twisting, and whole-body vibration for the entire working life of each subject [7]. A biomechanical analysis based on this expert assessment was conducted to determine the 'situational lumbar load' for each loading activity. Quantification was based on biomechanical model calculations while applying the three-dimensional dynamic simulation tool 'The Dortmunder' [8,9]. For the calculation of cumulative lumbar load (taking into account the whole working life), manual handling of objects of about 5 kg or more and postures with trunk inclination of at least 20 degrees or more are considered. Lumbar-disc compressive force is weighted overproportionally (squared) in relation to the respective duration of materials handling or intensive-load posture. We calculated the weight as mean body weight during adult lifetime prior to diagnosis; this mean was used to calculate BMI in terms of meters squared per kilogram. The cumulative smoking amount was calculated in terms of pack-years. Each sports activity was classified into one of four groups: endurance sports (jogging, biking, and swimming), ball sports (soccer, handball, volleyball, and basketball), athletics (apparatus gymnastics, shot put, javelin, hammer throwing, wrestling, and weight lifting), and body building sports (body building and strength training). The lifetime duration of sports activities was calculated for these four groups of sports activities.

### Categorization of variables

As an *a priori*-defined procedure, all metric independent variables were categorized in tertiles on the basis of the distribution of all exposed control subjects (men and women combined), the smallest category being the reference. If less than 20% of the control subjects were non-exposed, the reference category combined non-exposed subjects and subjects in the first exposure tertile. If the highest tertile of exposed control subjects comprised more than 10% of all control subjects (exposed plus non-exposed), a high-dose category was generated according to the 95th percentile of control subjects. In the case of BMI (to which all subjects were 'exposed'), tertiles were calculated including all controls, and a high-dose category comprising the upper 5% was defined.

### Characteristics of cases and control subjects

In Table 1, characteristics of the study populations are described. About half of the cases with lumbar disc herniation showed a radiculopathy with motor deficit (55% of male and 49% of female patients). Of male cases with symptomatic lumbar disc narrowing, 31% had a radiculopathy with motor deficit, 23% had a radiculopathy with sensory deficit, and 46% had a local lumbar syndrome with pronounced movement restriction (finger-to-floor distance of more than 25 cm). Of female cases with symptomatic lumbar disc narrowing, 25% had a radiculopathy with motor deficit, 19% had a radiculopathy with sensory deficit, and 54% had only a local lumbar syndrome with pronounced movement restriction. The mean age of patients with lumbar disc narrowing (55 years in men and 56 years in women) was higher than the mean age of control subjects. More men than women and more control subjects than cases had graduated from high school. The majority of men with lumbar disc disease had been exposed to the highest exposure tertile (according to the distribution of male and female control subjects combined) of cumulative lumbar load at work because of manual materials handling or intensive-load postures or both (67.1% of lumbar disc herniation cases and 69.7% of male lumbar disc narrowing cases had a workload of at least 13.26 meganewton-hours). In control subjects, the respective percentage was only 45.5. In women, too, the proportion of cases exposed to high cumulative workload was higher than in controls. Risk estimates for the relationship between physical workload and lumbar disc diseases are presented in an earlier publication [2].

**Table 1:** General characteristics of the study population

	Men						Women					
		
	Lumbar disc herniation (*n *= 286)		Lumbar disc narrowing (*n *= 145)		Control subjects (*n *= 453)		Lumbar disc herniation (*n *= 278)		Lumbar disc narrowing (*n *= 206)		Control subjects (*n *= 448)	
						
	N	%	N	%	N	%	N	%	N	%	N	%
Clinical symptoms in cases												
Radiculopathy with motor/sensory deficit	157	54.9	45	31.0			137	49.3	52	25.2		
Radiculopathy with sensory (and no motor) deficit	128	44.8	33	22.8			138	49.6	40	19.4		
Radiculopathy with motor or sensory deficit or both, not further classified	1	0.3	1	0.7			3	1.1	2	1.0		
Local lumbar syndrome	-	-	66	45.5			-	-	112	54.4		
Age at diagnosis/interview, years												
Less than 35	27	9.4	9	6.2	84	18.5	51	18.3	4	1.9	77	17.2
35 to less than 45	91	31.8	20	13.8	119	26.3	71	25.5	27	13.1	137	30.6
45 to less than 55	73	25.5	31	21.4	97	21.4	72	25.9	54	26.2	109	24.3
55 to less than 65	61	21.3	51	35.2	104	23.0	58	20.9	78	37.9	94	21.0
65 or more	34	11.9	34	23.4	49	10.8	26	9.4	43	20.9	31	6.9
Mean (standard deviation)	48.7 (11.1)		55.0 (10.7)		47.3 (12.6)		47.1 (11.8)		56.0 (9.8)		46.4 (11.8)	
Cumulative lumbar workload, MNh^a^												
Less than 1.59	19	6.6	7	4.8	68	15.0	98	35.3	62	30.1	232	51.8
1.59 to less than 13.26	75	26.2	37	25.5	179	39.5	82	29.5	58	28.2	121	27.0
13.26 or more	192	67.1	101	69.7	206	45.5	98	35.3	86	41.7	95	21.2
Educational level												
Graduated from high school	74	25.9	26	17.9	182	40.2	64	23.0	27	13.1	145	32.4
Secondary school level	83	29.0	32	22.1	125	27.6	101	36.3	59	28.6	173	38.6
Elementary level/no graduation	127	44.7	87	60.0	146	32.2	112	40.3	119	57.8	129	28.8
Unknown	2	0.7	-	-	-	-	1	0.4	1	0.5	1	0.2

^a^Cumulative lumbar workload refers to manual materials handling or intensive-load postures or both. MNh, meganewton-hours.

### Description of potential confounders and statistics

Odds ratios (ORs) and 95% confidence intervals (CIs) were calculated with an unconditional logistic regression analysis. All statistical analyses were conducted separately for men and women and were adjusted for age and study center, referred to as 'region' in this text. Age was entered into the logistic regression model in 10-year categories. In addition to the ORs adjusted solely for age and region, ORs for the 'final model' are presented. A set of all factors that might confound the relationship between the respective lifestyle variable and either lumbar disc herniation or lumbar disc narrowing (or both) was considered. Selection of confounders was done from the following list of biologically plausible factors: BMI, cumulative physical workload (including manually handled weights of at least 5 kg and trunk inclination of at least 20 degrees), pack-years, athletic activities (the four categories are described in the 'Exposure assessment' section above), whole-body vibrations, hip malformation, difference in length of legs, scoliosis of the lumbar spine, articular gout, Bechterew disease, Scheuermann disease, spondylolisthesis, malformation of the lumbar spine, fracture of the lumbar spine, psychosocial workload, and major life events (death of a spouse, severe disease, divorce, troubles in the family, or dismissal). Potential confounders were included in the final logistic regression model if they changed the OR of the considered lifestyle variable by more than 10% in at least one category. To calculate tests for trend, we included the specific exposures as continuous variables in the logistic regression model.

### Ethics

The study was regarded as quality development work within the area of occupational health. The aims, methods, and procedures of the study were agreed upon by the Hesse Medical Association. The study was performed in compliance with the ethical principles of the Declaration of Helsinki. The data protection guidelines were developed in cooperation with and agreed upon by the Hesse Federal Commissioner for Data Protection and Freedom of Information Decline. All subjects gave their informed consent.

## Results

### Association between lumbar disc disease and body mass index

ORs for risk of lumbar disc disease are presented in Table 2 for men and in Table 3 for women. We found generally higher risks of lumbar disc disease with increasing BMI in both sexes; tests for trend were statistically significant in both sexes for lumbar disc herniation and for lumbar disc narrowing. However, in men, there was no clear dose-response relationship for the risk of lumbar disc herniation in the categorized analysis, the highest risk being observed in men who were slightly overweight (BMI of 24.3 to less than 29.21) compared with those of the lowest BMI quartile (OR 2.6, CI 1.6 to 4.3). For lumbar disc narrowing, risk was increased in the second highest and highest categories (OR 1.3 and 2.6, respectively). Adjustment for additional confounders (workload for disc herniation, whole-body vibration, body building, and psychosocial stress at work for disc narrowing) attenuated the associations to some degree.

**Table 2:** Lifestyle factors and lumbar disc disease among men

					Lumbar disc herniation				Lumbar disc narrowing (chondrosis)					
						
	Control subjects		Cases						Cases					
											
	N	%	N	%	Adj. OR^a^	95% CI	Adj. OR^b^	95% CI	N	%	Adj. OR^a^	95% CI	Adj. OR^c^	95% CI
Body mass index														
<21.88	76	16.8	27	9.4	1.0	-	1.0	-	17	11.7	1.0	-	1.0	-
≥21.88 to <24.30	185	40.8	101	35.3	1.6	0.9-2.6	1.4	0.8-2.4	47	32.4	0.9	0.5-1.8	0.9	0.5-1.7
≥24.30 to <29.21	164	36.2	143	50.0	2.6	1.6-4.3	2.1	1.3-3.6	67	46.2	1.3	0.7-2.5	1.2	0.6-2.3
≥29.21	21	4.6	13	4.5	2.0	0.9-4.7	1.6	0.7-3.8	13	9.0	2.6	1.0-6.4	2.3	0.9-5.9
Trend test^d^					*P *= 0.001		*P *= 0.003				*P *= 0.09		*P *= 0.02	
Smoking, pack-years														
Never smoked	192	42.4	102	35.7	1.0	-	1.0	-	45	31.0	1.0	-	1.0	-
>0 to <8	68	15.0	34	11.9	1.0	0.6-1.7	1.0	0.6-1.7	17	11.7	1.3	0.7-2.5	1.3	0.7-2.6
≥8 to <20	86	19.0	63	22.0	1.3	0.8-1.9	1.2	0.8-1.9	32	22.1	1.6	0.9-2.7	1.6	0.9-2.8
≥20 to <40	69	15.2	70	24.5	1.7	1.1-2.6	1.6	1.0-2.5	33	22.8	2.0	1.1-3.4	1.9	1.1-3.3
≥40	36	7.9	16	5.6	0.8	0.4-1.5	0.8	0.4-1.5	18	12.4	1.4	0.7-2.7	1.4	0.7-2.7
Trend test^d^					*P *= 0.48		*P *= 0.66				*P *= 0.07		*P *= 0.07	
Endurance sports, cumulative hours														
0	178	39.3	122	42.7	1.0	-			74	51.0	1.0	-		
>0 to <1,300	77	17.0	41	14.3	0.9	0.6-1.4			10	6.9	0.4	0.2-0.8		
1,300 to <4,030	98	21.6	55	19.2	0.9	0.6-1.3			23	15.9	0.6	0.4-1.1		
4,030 to <9,880	75	16.6	45	15.7	0.8	0.5-1.3			24	16.6	0.8	0.4-1.3		
≥9,880	25	5.5	23	8.0	1.3	0.7-2.4			14	9.7	1.0	0.5-2.1		
Trend test^d^					*P *= 0.67						*P *= 0.81			
Ball sports, cumulative hours														
0	255	56.3	150	52.4	1.0	-			83	57.2	1.0	-		
>0 to <1,040	44	9.7	41	14.3	1.7	1.1-2.8			15	10.3	1.3	0.7-2.5		
1,040 to <2,700	72	15.9	36	12.6	0.9	0.5-1.4			21	14.5	1.0	0.5-1.7		
2,700 to <5,100	39	8.6	29	10.1	1.2	0.7-2.1			9	6.2	0.9	0.4-1.9		
≥5,100	43	9.5	30	10.5	1.1	0.7-1.9			17	11.7	1.3	0.7-2.4		
Trend test^d^					*P *= 0.65						*P *= 0.83			
Athletic sports, cumulative hours														
0	420	92.7	257	89.9	1.0	-			135	93.1	1.0	-	1.0	-
>0 to <500	10	2.2	10	3.5	1.7	0.7-4.2			1	0.7	0.4	0.04-3.0	0.4	0.05-3.6
500 to <1,560	10	2.2	11	3.8	1.8	0.7-4.4			6	4.1	1.8	0.6-5.3	2.1	0.7-6.5
≥1,560	13	2.9	8	2.8	1.1	0.4-2.8			3	2.1	0.7	0.2-2.6	0.8	0.2-2.8
Trend test^d^					*P *= 0.43						*P *= 0.66		*P *= 0.64	
Body building sports, cumulative hours														
0	395	87.2	257	89.9	1.0	-	1.0	-	136	93.8	1.0	-	1.0	-
>0 to <400	17	3.8	10	3.5	1.0	0.5-2.4	1.0	0.5-2.4	1	0.7	0.2	0.03-1.8	0.3	0.03-2.0
400 to <1,350	18	4.0	12	4.2	1.0	0.5-2.2	1.0	0.5-2.2	5	3.4	1.1	0.4-3.1	1.2	0.4-3.4
≥1,350	23	5.1	7	2.4	0.5	0.2-1.1	0.5	0.2-1.1	3	2.1	0.5	0.1-1.7	0.5	0.1-1.7
Trend test^d^					*P *= 0.16 (neg.)						*P *= 0.29 (neg.)		*P *= 0.30 (neg.)	

^a^Adjusted for age and region. ^b^Final model for lumbar disc herniation: Adjusted for age, region, and cumulative physical workload (OR for body mass index and smoking); adjusted for age, region, and differing leg length (OR for body building). ^c^Final model for lumbar disc narrowing: Adjusted for age, region, body building, whole-body vibrations and psychosocial workload (OR for body mass index); adjusted for age, region, and cumulative physical workload (OR for smoking); adjusted for age, region, unemployment as severe life event, and cumulative physical workload (OR for athletics); adjusted for age, region, and psychosocial workload (OR for body building). ^d^To calculate tests for trend, we included the exposure scores as continuous variables in the logistic regression model; (neg.) means *P *for trend for a negative association. Adj. OR, adjusted odds ratio; CI, confidence interval.

**Table 3:** Lifestyle factors and lumbar disc disease among women

							Lumbar disc herniation		Lumbar disc narrowing (chondrosis)					
								
	Control subjects		Cases						Cases					
											
	N	%	N	%	Adj. OR^a^	95% CI	Adj. OR^b^	95% CI	N	%	Adj. OR^a^	95% CI	Adj. OR^c^	95% CI
Body mass index														
<21.88	218	48.7	110	39.6	1.0	-	1.0	-	66	32.0	1.0	-	1.0	-
≥21.88 to <24.30	110	24.6	77	27.7	1.3	0.9-2.0	1.3	0.9-1.8	51	24.8	1.1	0.7-1.8	1.1	0.7-1.7
≥24.30 to <29.21	86	19.2	64	23.0	1.4	1.0-2.1	1.3	0.8-1.9	68	33.0	1.6	1.0-2.6	1.7	1.0-2.7
≥29.21	24	5.4	25	9.0	2.1	1.1-3.9	2.0	1.1-3.7	21	10.2	2.1	1.0-4.3	1.5	0.7-3.1
Trend test^d^					*P *= 0.006		*P *= 0.03				*P *= 0.001		*P *= 0.02	
Smoking, pack-years														
Never smoked	245	54.7	135	48.6	1.0	-	1.0	-	121	58.7	1.0	-	1.0	-
>0 to <8	90	20.1	52	18.7	1.1	0.7-1.7	1.0	0.7-1.6	25	12.1	0.8	0.5-1.4	0.8	0.5-1.4
≥8 to <20	56	12.5	49	17.6	1.7	1.1-2.6	1.5	1.0-2.4	30	14.6	1.5	0.9-2.7	1.5	0.8-2.5
≥20 to <40	48	10.7	34	12.2	1.3	0.8-2.2	1.0	0.6-1.7	25	12.1	1.0	0.6-1.8	0.8	0.5-1.5
≥40	7	1.6	6	2.2	1.6	0.5-4.9	1.4	0.4-4.5	4	1.9	1.0	0.3-3.6	0.7	0.2-2.7
Trend test^d^					*P *= 0.10		*P *= 0.47				*P *= 0.36		*P *= 0.93	
Endurance sports, cumulative hours														
0	194	43.3	118	42.4	1.0	-			103	50.0	1.0	-		
>0 to <1,300	100	22.3	65	23.4	1.1	0.7-1.7			25	12.1	0.8	0.4-1.3		
1,300 to <4,030	78	17.4	35	12.6	0.7	0.5-1.2			28	13.6	0.9	0.5-1.5		
4,030 to <9,880	55	12.3	39	14.0	1.2	0.7-1.9			30	14.6	1.1	0.6-1.8		
≥9,880	21	4.7	21	7.6	1.6	0.8-3.1			20	9.7	1.5	0.8-3.1		
Trend test^d^					*P *= 0.67						*P *= 0.54			
Ball sports, cumulative hours														
0	372	83.0	233	83.8	1.0	-			179	86.9	1.0	-		
>0 to <1,040	45	10.0	20	7.2	0.7	0.4-1.2			12	5.8	0.7	0.4-1.5		
1,040 to <2,700	21	4.7	18	6.5	1.4	0.7-2.8			9	4.4	1.2	0.5-2.8		
2,700 to <5,100	7	1.6	4	1.4	1.1	0.3-3.7			3	1.5	1.4	0.3-6.3		
≥5,100	3	0.7	3	1.1	1.7	0.3-8.7			3	1.5	4.3	0.6-29.4		
Trend test^d^					*P *= 0.65						*P *= 0.15			
Athletic sports, cumulative hours														
0	412	92.0	250	89.9	1.0	-			192	93.2	1.0	-	1.0	-
>0 to <500	11	2.5	7	2.5	1.2	0.4-3.0			3	1.5	0.7	0.2-2.6	0.8	0.2-3.1
500 to <1,560	14	3.1	9	3.2	1.0	0.4-2.4			7	3.4	1.1	0.4-2.8	1.2	0.4-3.3
≥1,560	11	2.5	12	4.3	1.8	0.8-4.3			4	1.9	1.5	0.4-5.5	1.4	0.4-5.4
Trend test^d^					*P *= 0.43						*P *= 0.74		*P *= 0.70	
Body building sports, cumulative hours														
0	408	91.1	249	89.6	1.0	-	1.0	-	202	98.1	1.0	-	1.0	-
>0 to <400	15	3.3	10	3.6	1.1	0.5-2.6	1.1	0.5-2.6	1	0.5	0.2	0.03-1.8	0.3	0.03-2.1
400 to <1,350	17	3.8	15	5.4	1.5	0.7-3.2	1.4	0.7-2.9	3	1.5	0.5	0.1-2.0	0.5	0.1-1.7
≥1,350	8	1.8	4	1.4	0.8	0.2-2.8	0.3	0.1-1.7	-	-	-	-	-	-
Trend test^d^					*P *= 0.94		*P *= 0.54 (neg.)				*P *= 0.12 (neg.)		*P *= 0.12 (neg.)	

^a^Adjusted for age and region. ^b^Final model for lumbar disc herniation: Adjusted for age, region, and cumulative physical workload (OR for body mass index and smoking); adjusted for age, region, and differing leg length (OR for body building). ^c^Final model for lumbar disc narrowing: Adjusted for age, region, body building, whole-body vibrations and psychosocial workload (OR for body mass index);adjusted for age, region, and cumulative physical workload (OR for smoking); adjusted for age, region, unemployment as severe life event, and cumulative physical workload (OR for athletics); adjusted for age, region, and psychosocial workload (OR for body building). ^d^To calculate tests for trend, we included the exposure scores as continuous variables in the logistic regression model; (neg.) means *P *for trend for a negative association. Adj. OR, adjusted odds ratio; CI, confidence interval.

In women, for the highest category, the association with disc herniation remained statistically significant even after cumulative workload was adjusted for. Association with lumbar disc narrowing approached statistical significance. Women with a BMI of at least 29.21 had more than twice the risk of disc herniation or disc narrowing than women with a BMI of less than 21.88. Adjustment for other confounders did not considerably change the results.

### Association between lumbar disc disease and smoking

There was no clear dose-response relationship between smoking amount and lumbar disc disease. Men with a 'medium' smoking amount (20 to less than 40 pack-years) showed significantly increased ORs for lumbar disc herniation and lumbar disc narrowing (OR 1.7 and 2.0, respectively). However, a very high smoking amount (at least 40 pack-years) was not associated with higher risk of lumbar disc herniation (OR 0.8, CI 0.4 to 1.5). In women, the highest risk occurred in those with 8 to fewer than 20 pack-years (OR for lumbar disc herniation 1.7, CI 1.1 to 2.6; OR for lumbar disc narrowing 1.5, CI 0.9 to 1.4). Selected confounders modified OR very little. Owing to small numbers of women who had consumed 40 pack-years or more, estimates for this high-risk group were imprecise with broad CIs.

### Association between lumbar disc disease and sports activities

For endurance sports, ball sports, and athletics (in men only), the OR did not change as a result of potential confounders by more than 10% in any category. Therefore, ORs for the final model differ only from the 'crude' ORs (adjusted solely for age and region) for body building (both sexes) and for athletics (women). In men, we found a decreased risk of lumbar disc narrowing for moderate levels of endurance sport activities (OR of between 0.4 and 0.8), and the risk returned to the baseline value in the highest exposure category. Highest levels of body building and strength training in men were non-significantly negatively associated with lumbar disc herniation and with lumbar disc narrowing: for a cumulative duration of 1,350 hours or more, the OR was 0.5 (CI 0.2 to 1.1) for lumbar disc herniation. In women, frequencies in high-level categories of most sports activities were too small to allow interpretation. Exposure to endurance activities of at least 9,880 hours (the highest category) was associated with about 50% higher risks of lumbar disc herniation (OR 1.6, 95% CI 0.8 to 3.1) or disc narrowing (OR 1.5, 95% CI 0.8 to 3.1).

## Discussion

In this multi-center case-control study, men and women with a BMI of at least 29.21 had about twice the risk of lumbar disc herniation and narrowing compared with those in the lowest category (BMI of less than 21.88). Adjustment for relevant confounders such as physical workload at work changed the risk estimates to only a small degree. No clear dose-response relationship was observed for smoking, and the highest risk was found in those exposed to moderate amounts of pack-years.

For a high cumulative amount of body building or strength training, we found non-significantly decreased risks for lumbar disc disease, although for women, low numbers of exposed subjects do not allow definitive conclusions. For endurance sports, ball sports, and athletics, no such preventive effect was found in either men or women.

Seidler and colleagues [10] reviewed the existing literature and did not find a clear relationship between body weight or smoking and lumbar disc disease. Four studies found significant associations between smoking and structural lumbar spine disease, whereas seven others did not. All in all, reviewed studies showed inconsistent results and were lacking in clear evidence of an association. Two epidemiological studies showed a statistically significant relationship between BMI and lumbar disc disease (disc herniation in men, but not in women, and spondylosis in both sexes). Six other studies revealed weaker but significant associations between weight and lumbar spine disease.

In a systematic review by Shiri and colleagues [11], the relationship of cardiovascular and lifestyle risk factors with lumbar radicular pain or clinically defined sciatica was investigated. Seven out of 13 studies, including cross-sectional, case-control, and cohort studies, confirmed an association between being overweight/obese and sciatic pain. Only one out of three studies found a dose-response relationship for this association [12]. Eleven studies investigated the role of smoking but had diverging results. In some occupational populations, an effect of long-term tobacco use on lumbar radiating pain was seen. The influence of physical activity was unclear, too, and some studies showed protective and others damaging effects on back pain.

The potential association between BMI and the risk of lumbar disc disease might be explained by both biomechanical and atherosclerotic processes. On the one hand, heightened physical load on lumbar discs caused by being overweight can lead to mechanical damage of lumbar discs. On the other hand, being overweight is associated with high levels of cholesterol and blood lipids which might enhance atherosclerotic processes in lumbar vessels and thus cause insufficient supply of lumbar discs. In necropsy studies included in a systematic review, Kauppila [1] found marked associations between atheromatous lesions in the aorta and lumbar disc degeneration and between stenosis of the feeding arteries of the lumbar spine and lower back pain during life. Epidemiological studies included in this review revealed associations of cardiovascular risk factors with disc degeneration and lumbar back pain. These results support the atherosclerosis hypothesis, while biomechanical damage due to weight pressure is consistent with the role of physical workload, due to the manual lifting of loads and trunk inclination, for lumbar disc disease. It is possible that both processes contribute to risk of lumbar disc diseases.

Despite the lack of a clear dose-response relationship, our data suggest a potential for reducing the burden of lumbar disc disease in the population by promoting healthy lifestyles, especially by reducing excess weight. According to Hennekens and Buring [13], the population-attributable risk (PAR) can be calculated as the proportion of exposed cases multiplied by (OR - 1)/OR. Accordingly, the PARs of lumbar disc herniation are about 30% in men for a BMI of at least 24.3 but only about 10% in women (mainly because of a lower prevalence of elevated BMI values).

### Strengths and limitations of the study

Unlike the majority of studies, the German EPILIFT study has the advantage of clinically confirmed diagnoses and a detailed assessment of exposure and covariates according to an *a priori*-defined analytic concept. The calculation of cumulative lumbar workload was based on expert assessment of occupational tasks and subsequent biomechanical analysis.

As a potential limitation of the study, the low participation rate (66% among cases and 53% among control subjects) might have introduced selection bias. According to a non-responder analysis, the proportion of blue-collar workers was higher in non-participating cases and control subjects, and this might be related to unfavorable lifestyle behavior. However, the non-responder analysis does not point to differential selection bias in cases and controls. Since radiographic examinations were available only for cases, the frequency of lumbar disc disease among the population controls is unknown. A presumed prevalence of up to 10% among population controls would result in a slight tendency to underestimate potential risk factors.

## Conclusions

According to our multi-center case-control study, being overweight might be related to lumbar disc herniation and to lumbar disc narrowing, but intervention studies particularly are needed to further clarify this relationship. Further studies should also deal with the potential protective role of body building or strength training in the occurrence of lumbar disc disease. Given the relatively high prevalence of overweight individuals (particularly males), a promising prevention strategy of lumbar disc disease therefore includes weight reducing in addition to organizational measures in work environment aimed at reducing the physical workload.

## Abbreviations

BMI: body mass index; CI: confidence interval; CT: computerized tomography; MRI: magnetic resonance imaging; OR: odds ratio; PAR: population-attributable risk.

## Competing interests

The authors declare that they have no competing interests.

## Authors' contributions

BS participated in the statistical analysis and drafted the manuscript. UB-A coordinated the study. AB and JG developed the diagnostic procedure. MJ performed the biomechanical lumbar-load analysis. RE developed and coordinated the expert assessments of physical work. GE and MM participated in the development of the standardized interview and interpretation of the study results. JH participated in the development of the epidemiological concept and in the interpretation of the study results. AS was responsible for epidemiological design, epidemiological analysis, and interpretation of data. All authors participated in the design and conduction of the study and read and approved the final manuscript.
